# Eliciting Psychological Ownership of Object by Marking Organizational Name: The Role of Belongingness

**DOI:** 10.3389/fpsyg.2021.699738

**Published:** 2021-10-25

**Authors:** Wenhua Wang, Jon L. Pierce, Dahui Li, Guangrong Wang, Jianbiao Li, Xiaofei Niu

**Affiliations:** ^1^Reinhard Selten Laboratory, Business School, China Academy of Corporate Governance, Nankai University, Tianjin, China; ^2^School of Economics, Institute for Study of Brain-Like Economics, Shandong University, Jinan, China; ^3^Department of Management Studies, University of Minnesota Duluth, Duluth, MN, United States; ^4^Neural Decision Science Laboratory, Weifang University, Weifang, China

**Keywords:** psychological ownership, organizational name, belongingness, P300, event-related potentials

## Abstract

Psychological ownership critically entails the need for home (a place in which to dwell or a place of belongingness). However, the question of how an individual’s need for belongingness within an organization affects their psychological ownership of organization-linked objects remains unexplored. We first conducted a behavioral study to determine whether psychological ownership of object can be elicited by marking the object with the name of the subjects’ organization. The participants in this behavioral study reported a higher level of psychological ownership when objects were marked with their own organization’s name (i.e., in-organization objects) compared with objects marked with another organization’s name (i.e., out-organization objects). Importantly, this effect was more pronounced among subjects who experienced a stronger sense of organizational belongingness. We subsequently conducted a second study to explore its underlying neural mechanism. Our findings indicated that participants with a higher level of perceived organizational belongingness exhibited a significantly larger amplitude of the P300 component of event-related potential in response to in-organization objects compared with their response to out-organization objects. However, no significant difference in the P300 component was found for participants who lacked a sense of organizational belongingness.

## Introduction

Psychological ownership is defined as a “state in which individuals feel as though the target of ownership is ‘theirs’ (i.e., it is MINE!)” (Pierce et al., 2001), and it is portrayed as both an affective and cognitive state that reflects an individual’s awareness, thoughts, and beliefs regarding the target object (Pierce et al., 2003).^1^ The target object can be sensed for anything, including tangible objects like cups or pens (Peck et al., 2013) and something rather intangible like a brand (Chang et al., 2015), a job (Brown et al., 2014), or an idea (Baer and Brown, 2012).

An important question is how to elicit the psychological ownership of a target object. The extant studies that focus on the consequences of psychological ownership mainly use the antecedents of controlling, investing the self, and intimately knowing to manipulate the level of psychological ownership, such as touching and imagining (e.g., Cunningham et al., 2008; Peck and Shu, 2009; Moreau et al., 2011). Moreover, it is suggested that self-object congruity facilitates the development of psychological ownership (Ye and Gawronski, 2016; Atasoy and Morewedge, 2018). The shared meaningful association between the self and the object, such as a Boston Red Sox fan and a “Red Sox” baseball hat, is likely to be a unique antecedent of psychological ownership (Morewedge, 2020).

With regard to self-object congruity, the previous research provides evidence that consumers value domestic products more as their own products than they value foreign products (e.g., Gineikiene et al., 2017) because there might exist a stronger self-object association and implicit self-object link with the domestic products than with the foreign products (Ye and Gawronski, 2016). Consumers attach shared meanings, cognitions, and emotions to in-group goods as they can serve as markers of their self-identities and socially recognized symbols (Belk, 1988; Dittmar, 1992). Consequently, the self-object link can be considered stronger for goods associated with one’s in-group comparing to those associated with the out-group (Dommer and Swaminathan, 2013). Such self-object link is one of the fundamental conceptual cores of psychological ownership, reflecting the fact that an individual is being psychologically tied to the target and owned objects and that the individual often views the object as the extension of one’s individual self (Pierce and Peck, 2018). In a similar vein, we suggest that objects marked with the name of an individual’s organization (i.e., in-organization objects), rather than objects with the name of another organization (i.e., out-organization objects), become embedded in the individual’s self-presentation and self-cognition so that the self-object link is enhanced. Thus, we first hypothesize that the individual will perceive higher psychological ownership of in-organization objects than that of out-organization objects.

In addition, the psychology literature suggests that people are driven to engage in activities that satisfy their specific psychological needs (e.g., see Ryan and Deci, 2000). A need for a sense of home, a place to dwell, or a space of belonging in the world has been elaborated as a root of psychological ownership (Pierce and Jussila, 2011). Belongingness serves the individual’s basic need for “having a place.” Although the “place” is sometimes a geographic concept, it can also be referred to as a social community, such as an organization (Porteous, 1976; Pierce et al., 2003). The need for belongingness motivates individuals to claim control of their surroundings and invest their selves into the organization. When people perceive themselves to be owners within an organization, their need for belongingness is satisfied (Avey et al., 2009). This feeling provides the owner with psychic security and comfort, as well as a sense of control and identity (Van Dyne and Pierce, 2004). Furthermore, the satisfaction of the need for belongingness may further contribute to the congruity between the self and the organization (Walasek et al., 2015). Therefore, the sense of being psychologically attached to the organization and its related objects can be facilitated among individuals who have a stronger sense of organizational belongingness. Based on the analysis above, we hypothesize that perceived psychological ownership will be moderated by organizational belongingness, such that individuals who have a strong sense of organizational belongingness will perceive higher psychological ownership of in-organization objects compared with individuals who lack a perception of organizational belongingness.

In addition to the identification of the above-mentioned boundary conditions, however, the cognitive mechanisms that support the development of psychological ownership have not been so far explored. Researchers attempt to explore different aspects of psychological ownership using reaction time-based measures, such as the implicit-association test (see Gawronski et al., 2007; Ye and Gawronski, 2016; LeBarr and Shedden, 2017). Such measures have advantages over self-reported measures that are often seen in the social psychological literature because these measures reveal less controlled aspects of attitudes that unfold relatively quickly (Lust and Bartholow, 2009). In this study, we introduce the method of event-related potentials (ERP) to examine psychological ownership. The high temporal resolution of the ERP technique represents a significant advantage in studying the dynamic development of psychological ownership, as the method could reveal the response time of the brain to stimuli in milliseconds (Tivadar and Murray, 2019).^2^

Several neuroscientific studies have explored the neural basis of psychological ownership. For example, Turk et al. (2011) asked their subjects to imagine placing an object in a basket that was theirs (e.g., “Put the ball in your basket”) and subsequently perform an object judgment task. They found that self-ownership cues were associated with increased attentional processing measured as the P300 component. However, psychological ownership in their experiment was elicited passively through an associative-learning task instructed by the experimenter. Consequently, it remains uncertain whether psychological ownership motivated by the fulfillment of basic human needs, such as belongingness, involves a prioritized access to the brain and its associated cognitive areas that participate in the processing of relevant external stimuli of objects that belong to different groups (in-organization vs. out-organization). That is, are objects that are perceived to be psychologically owned due to self-object link or extended self, rather than manipulated as psychologically owned shortly during the conduct of an experiment, preferentially attended to by experimental subjects, despite the subjects’ participation in an experiment?

Here, we aim to gain further insights into the temporal dynamic of neural mechanisms involved in psychological ownership that is elicited by marking objects with the name of subjects’ organization as well as the role of perceived organizational belongingness in relation to psychological ownership. As shown by Turk et al. (2011) and Muñoz et al. (2020), P300 is one major ERP component related to the link between the self and an owned object. The P300 component, which is characterized by a large positive wave around the 300–500 milliseconds (ms) time point following stimulus presentation, is commonly maximal in amplitude at the parietal electrodes. Its amplitude is proportional to the allocation of attentional resources (see Polich, 2007, for a review), and it is not influenced by factors related to the selection or execution of responses (Gray et al., 2004). In the present study, in-organization objects may be perceived by individuals as being their “own” to a greater extent than out-organization objects due to self-object congruity. Such a strong association between the self and the object based on self-presentation potentially underlies the perceptual and attentional salience of an individual (De Bortoli Vizioli et al., 2020), which could be reflected in the P300 component. Therefore, we hypothesize that the P300 elicited by in-organization objects will have a larger amplitude than that elicited by out-organization objects. Considering the role of belongingness and its role in the development of psychological ownership, we also expect that there will be a greater difference in P300 among subjects who have a strong sense of organizational belongingness when these subjects are presented with the two types of objects (in-organization vs. out-organization).

The present study has two main purposes. The first is to understand the role of organizational belongingness plays in shaping individuals’ psychological ownership of in-organization objects versus those with the name of another organization (i.e., out-organization objects). The second is to explore the time course of neural activity evoked by such ecological psychological ownership cues as well as the association between neural measures and the conventional attitudinal measures of psychological ownership. To this end, we used a perceptual task, in which participants had to indicate their psychological ownership of an object (either in-organization or out-organization) as well as organizational belongingness. We then recorded the temporal dynamics of the brain activity relative to the processing of these two types of objects. As electrophysiological measures may reveal differences that attitudinal measures are not sensitive enough to detect (Williams et al., 2016), we were able to compare the neural evidence during identifications of different objects by individuals with varying level of organizational belongingness.

## Study 1

The goal of Study 1 was to examine whether marking objects with the name of an organization would elicit psychological ownership. We also tested the role of perceived organizational belongingness in eliciting the psychological ownership of objects. For these purposes, we collected attitudinal data *via* a platform called “Wenjuanxing” (meaning “survey star” in Chinese) in mainland China, which provides functions equivalent to Amazon Mechanical Turk. Study 1 was carried out after the approval of the Ethics Committee of Nankai University.

### Methods

#### Participants

A total of 440 students (100 men, age range: 17–25, mean age=19.89years, SD=1.39) participated in the study. They were undergraduate students of Nankai University Binhai College.

#### Stimuli

**Figure 1:**
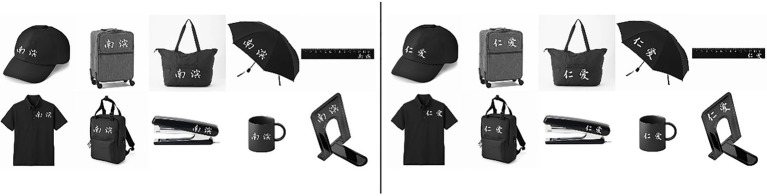
Stimuli used in the study. The images on the left side depict in-organization objects, which were marked with the name of the subjects’ own organization “南滨” (i.e., NanBin). The images on the right side depict out-organization objects marked with the name of the other organization “仁爱” (i.e., Ren’ai).

The stimuli used in this study were images of 10 different objects that were very common commodities available for purchase in any large offline/online market (e.g., mugs, umbrellas, and hats; see Figure 1). We marked these objects with either the name of the participants’ college (i.e., Nankai University Binhai College, abbreviated as “NanBin”) or the name of another college (i.e., Tianjin University Ren’ai College, abbreviated as “Ren’ai”). Both colleges were located in Tianjin, China, but they were affiliated with different universities. In this way, we made a distinction between in-organization objects (objects marked with “NanBin”) and out-organization objects (objects marked with “Ren’ai”). All of the pictures had the same format (400×400 pixels). All other physical properties of the images were identical except for the objects presented.

#### Procedures

The subjects were initially asked to read a brief introductory passage about their college, including its mission statement, strengths, and honorary titles. Then, they were asked to complete a questionnaire that measured their sense of organizational belongingness relating to their own organization, namely, NanBin. Next, the subjects were asked to observe the images of the in-organization and out-organization objects carefully and to confirm they were familiar with the details of these objects. As a final step, they responded to a psychological ownership scale to measure their self-reported degree of psychological ownership of objects with different organization’s name. We also recorded their grade and whether they served as student cadres or not.

#### Measures

##### Sense of Organizational Belongingness

We applied the Psychological Sense of School Membership Scale (PSSM) by Goodenow (1993), which was a six-point scale (1 = “strongly disagree” and 6 = “strongly agree”). We used 18 items to measure a subject’s sense of organizational belongingness. Sample items included the following: “I feel like a real part of (name of the university),” “I feel proud of belonging to (name of the university),” and “I am included in lots of activities at (name of the university).” Each subject’s total PSSM score, which ranged from 0 to 108, measured their sense of organizational belongingness, with higher scores corresponding to a stronger sense of belongingness. Cronbach’s *α* was 0.82.

##### Psychological Ownership

The participants responded to a six-item scale developed by Fuchs et al. (2010) and validated by Van Dyne and Pierce (2004) and by Peck and Shu (2009) that measured their psychological ownership of objects marked with the name of the organization. Examples of items included “I feel that these objects belong to me” and “Although I do not legally own the object, I have the feeling that the object is mine.” Their responses were scored with a seven-point Likert scale ranging from 1=“strongly disagree” to 7=“strongly agree.” The subjects’ scores for the psychological ownership scale ranged from 0 to 42, with higher scores corresponding to higher levels of self-reported psychological ownership. Cronbach’s *α* for the psychological ownership scale was 0.87.

### Results

Based on the median organizational belongingness score (81), we divided participants into two groups, that is, high belongingness group (hereafter HBG) and low belongingness group (hereafter LBG). Each of these groups had 214 participants, with the remaining 12 participants who scored 81 (the median belongingness score) were excluded from belongingness group comparisons.^3^ The average score for organizational belongingness in the HBG was 92.60 (SD=7.67), while that for the LBG was 69.85 (SD=8.83).

We carried out a 2×2 repeated measures ANOVA on self-reported psychological ownership scores with Group (HBG, LBG) as between-subject factor and object type (in-organization object, out-organization object) as within-subject factor to assess the effects of organizational belongingness and object type on psychological ownership.

There was a main effect of object type on self-reported psychological ownership [*F* (1,426)=790.78, *p*<0.001, $\eta_{p}^{2}$ = 0.65]. This result revealed that overall, subjects perceived a higher level of psychological ownership over in-organization objects than they did over out-organization objects. The main effect of the belongingness group was also significant [*F* (1,426)=184.21, *p*<0.001, $\eta_{p}^{2}$ = 0.085]. Meanwhile, a significant group × object type interaction was observed [*F* (1,426]=83.21, *p*<0.001, $\eta_{p}^{2}$ = 0.16]. A simple effect test showed that in-organization objects evoked a stronger degree of psychological ownership compared with out-organization objects in both groups [HBG: *F* (1.58)=693.50, *p*<0.001; LBG: *F* (1.58)=180.48, *p*<0.001]. Importantly, in-organization objects elicited stronger psychological ownership in the HBG than they did in the LBG [*F* (1.58)=338.33, *p*<0.001]. However, no group difference was found for the out-organization object [*F* (1.58)=115.15, *p*=0.159].

**Figure 2:**
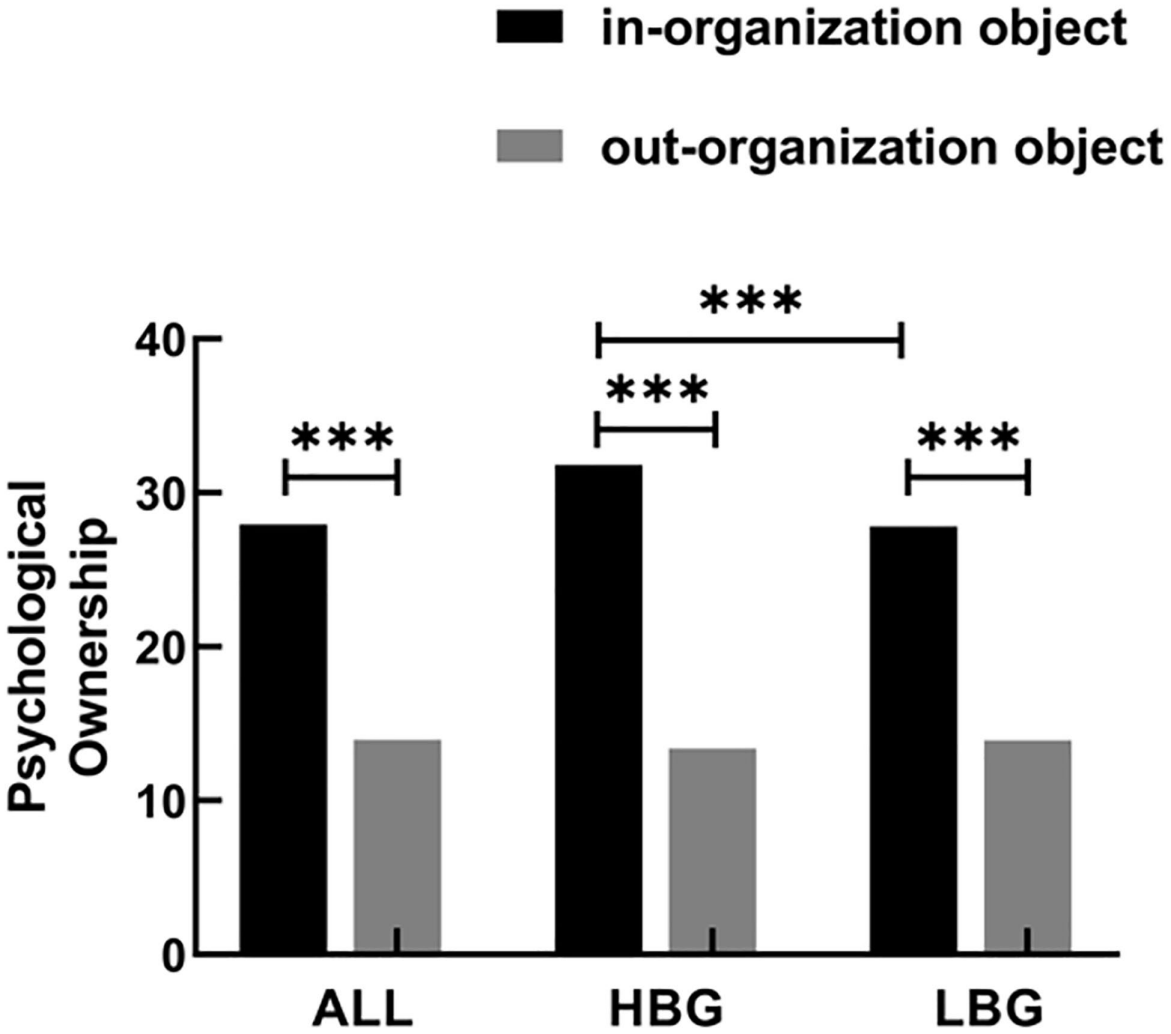
Average scores for participants’ psychological ownership in study 1. “∗∗∗” represents that *p*<0.01. Larger values on the vertical axis indicated stronger feelings of psychological ownership (range: 0–42). Self-reported psychological ownership scores for in-organization objects were significantly higher than those for out-organization objects.

Similarly, the paired t-test results of subjects’ psychological ownership scores for two kinds of organization-linked objects within the whole sample suggested that subjects perceived higher level of psychological ownership over in-organization objects (M=27.95, SD=8.69) than they did over out-organization objects (M=13.96, SD=7.65; *t*=26.06, *p*<0.001; see Figure 2). There was a significant positive correlation between the subjects’ sense of organizational belongingness and their psychological ownership of in-organization objects (*r*=0.59, *p*<0.001), thus proving that the state of belongingness and its association with home served as a motive driving the emergence of psychological ownership.

### Discussion

The results of Study 1 revealed that subjects perceived a higher level of psychological ownership over in-organization objects than they did over out-organization objects. This effect was more pronounced for the subjects with stronger sense of organizational belongingness, as a significant higher level of psychological ownership over in-organization objects in the HBG than in the LBG was found. But one concern of Study 1 was that we did not control for the subjects’ sense of belongingness in relation to the other organization. We addressed this concern in Study 2.

## Study 2

The main goal of Study 2 was to explore the time course of brain activity evoked by different psychological ownership cues, namely, in-organization or out-organization objects. In addition, the subjects’ sense of belongingness to an organization other than their own was controlled to assess the robustness of the findings of Study 1. Study 2 was carried out in accordance with the recommendations of the Ethics Committee of Nankai University.

### Methods

#### Participants

We recruited additional 76 undergraduate students from Nankai University Binhai College. Based on the sense of belongingness score, 30 subjects with high sense of belongingness to their organization (HBG) and 30 subjects with low sense of belongingness (LBG) were invited to participate the ERP study.^4^ Scores for the organizational belongingness of subjects in the HBG were significantly higher than those of subjects in the LBG (paired t-test: *p*<0.001, HBG: M=103.67, SD=5.17, LBG: M=61.74, SD=6.83). Three subjects in the LBG and one subject in the HBG were excluded because of technical problems and artifacts in the electroencephalogram (EEG) data. The brain activities of 27 subjects (*n*=27; 9 men, age range: 18–23, mean age=19.63years) in the LBG and of 29 subjects (*n*=29; 10 men, age range: 17–22, mean age=19.4years) in the HBG were fully analyzed.

We also collected data on the subjects’ sense of belongingness in relation to the other organization (i.e., Tianjin University Ren’ai College). Scores for belongingness relating to this other organization were similar for participants in the HBG and the LBG (paired *t*-test: *p*=0.164, HBG: M=45.57, SD=3.39, LBG: M=47.07, SD=4.86).

All subjects were right-handed and native Chinese speakers with normal or corrected-to-normal vision. No subjects reported the history of psychiatric or neurologic disorders. All subjects were provided written, informed consent forms for their participation and received a base payment of 50 Chinese yuan (approximately US $8.00) as compensation.

#### Stimuli and Procedures

The stimuli in this study were the same with Study 1. We ruled out the effect of stimulus familiarity. The degree of familiarity with the two types of objects was rated by subjects on a 7-point scale (1=“not familiar at all” to 7=“extremely familiar”). The degree of familiarity was reported to be 6.45 (SD=0.14) for in-organization objects and 6.36 (SD=0.15) out-organization objects. The paired t-test results showed that there were no significant differences between the different organization-linked objects in terms of their perceived familiarity (*t*=0.43, *p*=0.82). To prevent the effect of habituation, the order in which the stimuli were presented was pseudorandomized, so that the same type of object was not presented more than two times consecutively.

**Figure 3:**
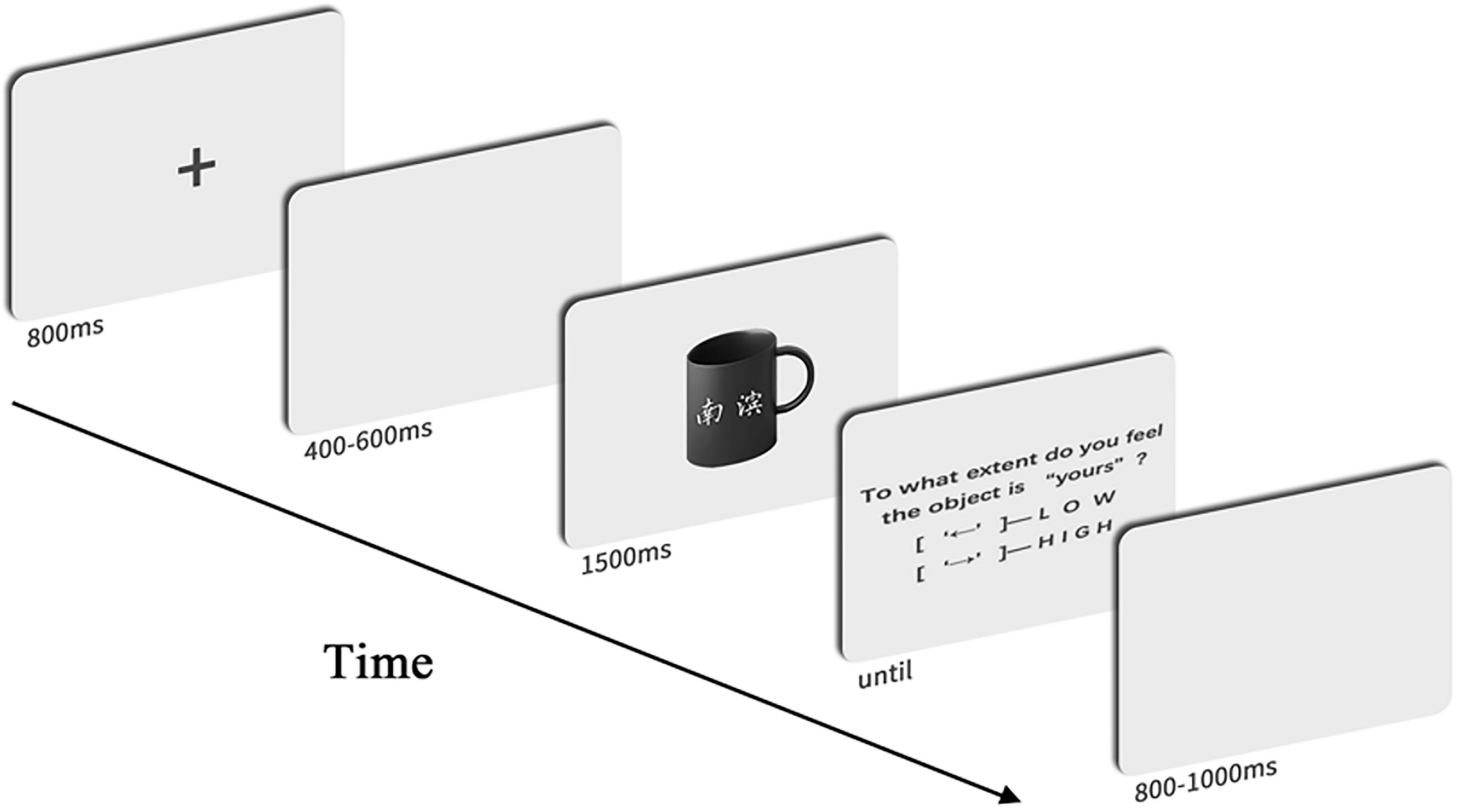
Time course of a single trial. Each trial began with an 800-ms fixation point followed by blank screen, which were randomized between 400 and 600ms. A screen displaying the object was shown for 1,500ms. Then, a question screen appeared until the participants responded. The inter-trial interval was randomized to last between 800 and 1,000ms.

EEG recordings were carried out in a sound-attenuated and electrically shielded chamber. Following the attachment of the electrodes on their heads, the subjects were seated in a comfortable chair which was placed approximately 100cm in front of a 23-inch computer monitor. Before beginning the task, subjects carefully read the instructions and were asked to participate in one or more five-trial practices until they understood the task. Figure 3 illustrates the time course of a single trial. Each trial began with the presentation of a single, centrally located white cross for fixation for 800ms. A blank screen was then presented for 400–600ms, followed by the presentation of a picture of an in-organization or out-organization object located in the center of the screen for 1,500ms. After recording the ERP, the following question appeared on the screen: “To what extent (low or high) do you feel that the object is yours?” The question remained on the screen until the subject pressed a button on a two-key response pad. There were two options for responding to the question in counterbalanced trials (left=“low” and right=“high” or right=“low” and left=“high”). At the end, the participants also responded to the Fuchs et al. (2010)‘s scale to measure their self-reported psychological ownership relating to different objects.

The entire experiment comprised a minimum of five practice trials and 100 test trials (two blocks of 50 trials). Only the latter test trials were used for ERP analysis. Each block was separated by a break, the duration of which was determined by the subject. All 100 trials were performed within 15–25min, with the trials randomly presented. E-Prime software v2.0 (Psychology Software Tools, Sharpsburg, PA, United States) was used to control the display of stimuli and for data acquisition.

#### EEG Acquisition and Analysis

Continuous EEG recordings were carried out with a 40-channel NuAmps DC amplifier (Compumedics Neuroscan, Charlotte, NC, United States). We used 32 active Ag/AgCl electrodes according to the International 10–20 System. The impedance of each electrode was maintained at <10kΩ. The reference and ground electrodes were placed at AFz. Electrodes below and above the left eye, as well as those located on the outer canthi of each eye, measured bipolar vertical and horizontal electrooculogram activities. Online EEG signals were digitized at a sampling rate of 1,000Hz using a 22-bit A/D converter.

EEG data were preprocessed using the EEGLAB v13.5.4 in MATLAB 2013b (MathWorks, Natick, MA, United States). The reference of EGG signals was reset to the average of the left and right mastoids, and a 1/30-Hz high−/low-pass filter was applied. Individual epochs were extracted from −200 to 1,000ms around the response. Epochs with an EEG amplitude exceeding ±100μV on any channel were rejected as artifact. An independent component analysis (ICA) was performed to remove eye movement, and the ICA components related to eye movement were manually selected. Artifact-free ERP trials were averaged separately for each experimental condition. Subjects had no fewer than 40 artifact-free epochs in each condition.

Clean EEG data were analyzed in the time domain. The 1,000-ms epochs were extracted starting at 200ms before subjects saw the stimuli. A 200-ms pre-seeing period was used as baseline, and the accepted epochs were baseline-corrected. We analyzed the peak amplitudes of the P300 across different sets of electrodes according to ERP topographies and relevant literatures. The P300 was defined as the peak amplitude occurring 300–500ms after the onset of object presentation at the electrode sites of Pz, P3, and P4.

A three-way repeated measure ANOVA was carried out for all measures of latencies and for the amplitudes relating to each component. The corresponding ANOVA factors were group (two levels: HBG and LBG), stimulus type (two levels: in-organization object and out-organization object), and laterality (three levels: left, midline, and right sites). For all ANOVAs, *p*-values were corrected using the Greenhouse–Geisser correction when the sphericity assumption was violated; *p*<0.05 was considered significant. Significant interactions were analyzed with the simple effect model. The t test with Bonferroni correction for multiple comparisons was used for *post hoc* analyses. Statistical analyses were performed using SPSS v19.0 (IBM, Armonk, NY, United States).

### Results

#### Behavioral Results

We carried out a 2×2 repeated measures ANOVA on self-reported psychological ownership scores as in Study 1. The main effects of object type [*F* (1.58)=466.07, *p*<0.001, $\eta_{p}^{2}$ = 0.89] and participants’ perceptions of organizational belongingness were significant [*F* (1.58)=184.21, *p*<0.001, $\eta_{p}^{2}$ = 0.76]. Moreover, the group × object type interaction [*F* (1.58)=182.64, *p*<0.001, $\eta_{p}^{2}$ = 0.76] was also significant. The results of a simple effect test revealed that in-organization objects evoked a higher degree of psychological ownership than out-organization objects in both groups of subjects [HBG: F (1.58)=616.12, *p*<0.001; LBG: F (1.58)=32.60, *p*<0.001]. Further, in-organization objects elicited a higher degree of psychological ownership in the HBG (M=36.90) than they did in the LBG (M=16.03; *F* [1,58]=338.33, *p*<0.001). However, no group difference was found for the out-organization object [*F* (1.58)<0.01, *p*=1; HBG: M=9.8, LBG: M=9.8]. Furthermore, a significant positive correlation was found between the subjects’ sense of belongingness and measures of psychological ownership relating to in-organization objects (*r*=0.93, *p*<0.001), further verifying the robustness of the findings of Study 1.

During the EEG recordings, 86.21% (24/29) of the subjects in the HBG selected “3” (i.e., a high degree of ownership) for in-organization objects in all of the trials. The remaining subjects selected “1” (i.e., a low degree of ownership) in no more than five trials. Further, 66.67% (18/27) of the subjects in the LBG selected “3” in all of the trials, and five subjects selected “1” in more than 10 trials. For out-organization objects, 79.31% (23/29) of the subjects in the HBG selected “1” in all of the trials, 62.96% (17/27) of the subjects in the LBG selected “1” in all of the trials, and four subjects selected “3” in more than 10 trials.

The reaction times were evaluated by 2×2 repeated measures ANOVA with group (HBG vs. LBG) and object type (in-organization vs. out-organization) as the two factors. No significant effects were observed [*F* (1.54)=0.07, *p*=0.80 and *F* (1.54)=0.03, *p*=0.87, respectively]. Moreover, there was also no significant interaction effect between group and object type [*F* (1.54)=0.46, *p*=0.50].

#### ERP Results

**Figure 4:**
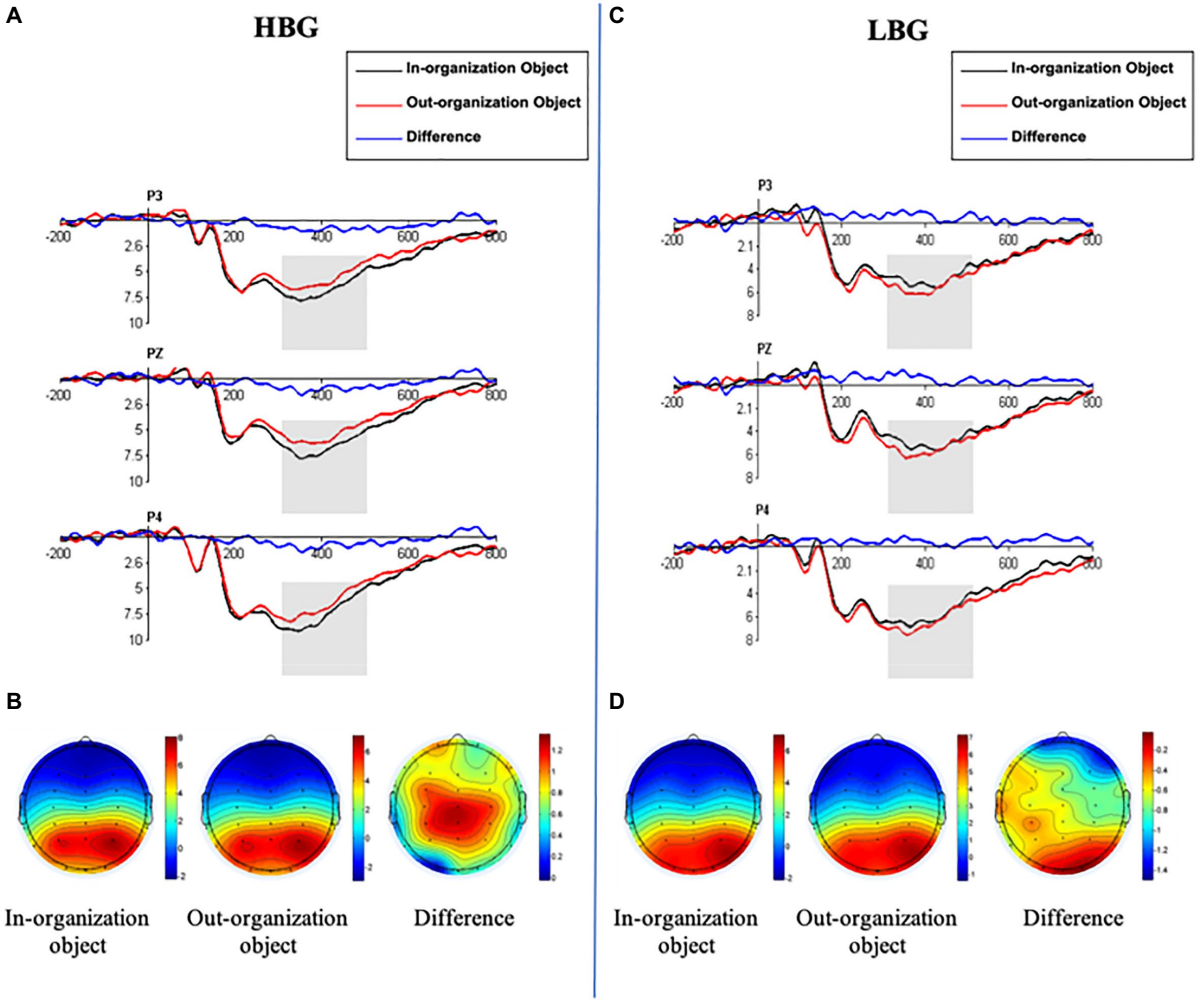
Event-related potentials (ERP) results for Study 2. **(A,C)** The Grand mean of ERP waves computed at a set of frontal-central sites (P3, Pz, and P4) of subjects in the HBG **(A)** and in the LBG **(C)**. **(B,D)** Topographic voltage maps of mean amplitudes of P300 (300ms–500ms) for subjects in the HBG **(B)** and in the LBG **(D)**.

Grand average ERP waveforms over three electrodes (Pz, P3, and P4) were presented in Figure 4. In-organization objects triggered a more positive-going deflection (P300) than out-organization objects for subjects in the HBG. In contrast, this pattern appeared to be reversed for subjects in the LBG. The out-organization objects elicited a slightly more positive-going P300 component within the same time window.

ANOVA of P300 amplitudes showed that neither the main effect for group [*F* (1.54)=2.82, *p*=0.099, $\eta_{p}^{2}$ = 0.05] nor that of object type [*F* (1.54)=0.40, *p*=0.53, $\eta_{p}^{2}$ = 0.007] was significant. However, there was a significant interaction effect between object type and group [*F* (1.54)=16.78, *p*=0.001, $\eta_{p}^{2}$ = 0.24]. Simple effects ANOVA revealed that the in-organization objects elicited greater P300 amplitudes for the subjects in the HBG (M=9.82μV) than in the LBG [M=7.73μV; *F* (1.54)=8.13, *p*=0.006]. However, no group difference was found for the out-organization objects [*F* (1.54)=0.19, *p*=0.66; HBG: M=8.81μV, LBG: M=8.47μV]. Moreover, in-organization objects evoked greater P300 amplitudes compared with out-organization objects [*F* (1.54)=11.60, *p*=0.001] for the subjects in the HBG, whereas no significant difference was observed for object types in the LBG [*F* (1.54)=6.79, *p*=0.060]. Additionally, there was a significant main effect of laterality [*F* (2,108)=19.11, *p*<0.01, $\eta_{p}^{2}$ = 0.26]. *Post ho* tests showed that the scalp right region (M=9.56μV) had a larger P300 amplitude than did the left (M=8.20μV) and middle (M=8.37μV) regions (Figure 4).

**Figure 5:**
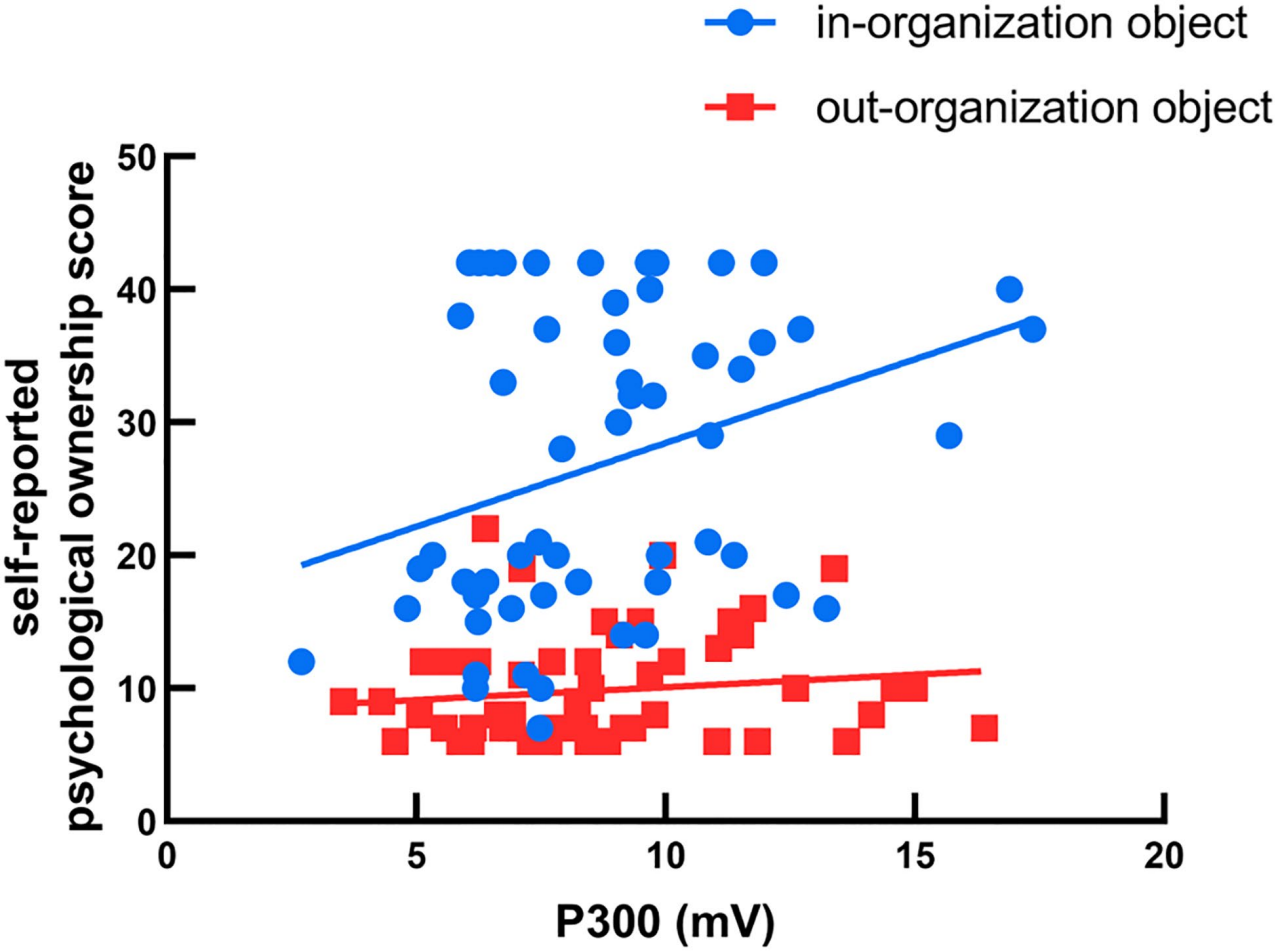
Correlation between subjects’ self-reported psychological ownership and the amplitude of P300. Self-reported psychological ownership was calculated by scales, and the P300 was defined as the peak amplitude occurring 300–500ms after the onset of object presentation at the electrode sites of Pz, P3, and P4.

Neither the main effect for object type nor its interaction with either group was significant for P300 latencies (*p*>0.05). On the other hand, the main effect of laterality on P300 latencies [*F* (2,108)=13.42 *p*<0.001, $\eta_{p}^{2}$ = 0.199] was significant. *Post hoc* test results revealed that shorter latencies had a stronger effect on the right region (M=360.39ms) than on the left (M=372.28ms) and midline regions (M=389.25ms), demonstrating that P300 could be used to predict the level of psychological ownership over in-organization objects. In addition, we tested the correlations between subjects’ self-reported psychological ownership (indicated by scales) and the amplitudes of P300 (indicated by the P300 amplitude between 300 and 500ms post-target image onset, over P3, Pz, and P4 electrode sites). We found a significant positive relationship between psychological ownership and P300 for in-organization objects (*r*=0.323, *p*=0.015), whereas no significant correlation was found for out-organization objects (*r*=0.136, *p*=0.319; Figure 5). We also found a significant positive relationship between the difference of P300 (calculated by subtracting the P300 amplitude for the out-organization object from the P300 amplitude for the in-organization object) and the self-reported psychological ownership of the in-organization object (*r*=0.52, *p*<0.001).

### Discussion

The behavioral results of Study 2 were consistent with Study 1. Importantly, the results of Study 2 indicated that among subjects in the HBG, significantly larger P300 amplitudes were generated in response to in-organization objects than for out-organization objects, whereas this effect was not evident among subjects in the LBG. P300 could thus be used as a neural index of psychological ownership derived from the fulfillment of the need for belongingness, with a larger amplitude reflecting a stronger sense of ownership.

## General Discussion

In the present study, we examined psychological ownership of objects elicited by marking them with the name of the subjects’ organization and tested its underlying behavioral and neural mechanisms. In Study 1, we collected participants’ behavioral data and found that marking objects with the name of the participants’ own organization could elicit psychological ownership of the objects. Importantly, subjects with a strong sense of organizational belongingness perceived a significantly higher level of psychological ownership of objects marked with their organization’s name compared with subjects with a weaker sense of belongingness. We subsequently conducted an ERP study (Study 2) to provide neural evidence for the role of organizational belongingness in eliciting the psychological ownership of in-organization objects. The ERP results showed that subjects with a strong sense of organizational belongingness generated a significantly larger P300 amplitude in response to the objects marked with their own organization’s name compared with the P300 amplitude that they generated in response to objects marked with another organization’s name. However, no significant difference in P300 amplitudes was observed for subjects with a weak sense of organizational belongingness. We thus confirmed that P300 could be a neural index of psychological ownership.

On the behavioral level, several laboratory experiments (Cloutier and Macrae, 2008; Cunningham et al., 2008; Kim and Johnson, 2012; Turk et al., 2013; Lebarr and Shedden, 2017; Lockwood et al., 2018) have validated the theory of psychological ownership. It is shown that subjects’ psychological ownership can influence their evaluations of the self and of the target (e.g., Gawronski and Bodenhausen, 2007; Dommer and Swaminathan, 2013), as well as their judgment and memory (e.g., Cloutier and Macrae, 2008; Cunningham et al., 2008; Turk et al., 2013). However, most of these studies used the antecedents of control, investing the self, and intimately knowing (Pierce et al., 2001) to experimentally manipulate the feeling of ownership, for example, through touching and imagining (Cunningham et al., 2008; Peck and Shu, 2009; Moreau et al., 2011). Unlike these earlier studies, the present study elicited different perceptions of psychological ownership by marking objects with the name of subjects’ own organization or another organization. Such personally meaningful objects characterize psychological ownership that is derived from the satisfaction of basic human needs, such as finding a “second home” within organization.

Psychological ownership is thought to stem from certain psychological roots or motives, one of which is the need for being at home within (Pierce et al., 2003). The construct “home” (dwelling) can be seen as the anchoring of one’s self in time and space, for example, a certain language, food, or physical place. Our results provided empirical evidence for this theoretical perspective. For undergraduates who were participants in our first experiment, they all “inhabited” their college, which was also an organization. However, satisfaction of the “need for belongingness” varies from person to person. Individuals with a stronger sense of organizational belongingness are more likely motivated to invest their time, effort, and self into the organization, to exercise control over the organization, and to develop a deeper knowledge of the organization (Karahanna et al., 2015). The congruity between the self and the organization is also enhanced, thereby facilitating the sense of being psychologically attached to the organization and its related objects. The above reason accounts for our finding that the participants in our study perceived a higher level of psychological ownership over in-organization objects than they did over out-organization objects, and this difference was even more significant for the subjects with a strong sense of organizational belongingness.

The ERP study revealed that cues associated with a high level of psychological ownership (marking an in-organization object) generated a stronger P300 response than did other cues among subjects who possessed a strong sense of organizational belongingness. According to the work of Miyakoshi et al. (2007), the differentiation of objects belonging to oneself from those belonging to others corresponds to the activation of the P300 component. Turk et al. (2011) further found that self-ownership cues were associated with increased attentional processing, measured as the P300 component, even when the basis of ownership was arbitrary and transient. More recently, Xu et al. (2017) observed that more early attention resources, as revealed by an enhanced P300 amplitude, were devoted to discriminate highly important self-related content from minimally important content, indexing an increased attentional processing when the subject evaluated an object to be self-related. Consistent with these findings, we found that the P300 amplitude generated by in-organization objects was larger than that generated by out-organization objects, because subjects regarded in-organization products more as their “extended self” than they did out-organization objects. In terms of the lateralization of P300, we found that the scalp right region had a larger P300 amplitude than did the left and middle regions. Consistent with previous studies, we demonstrated the association between self-referential processing and right-lateralized brain activities (Northoff et al., 2006; Esslen et al., 2008; Beer and Harris, 2019). The right hemisphere, the right superior frontal and inferior parietal cortex, in particular, played a predominant role in information processing that was related to self (Legrand and Ruby, 2009). These findings suggest that the time course of neural activity evoked by psychological ownership associated with the intrinsic motivation of belonging is to some extent approximates that of experimentally defined temporary psychological ownership. Interestingly, there was a greater difference in P300 amplitude relating to two different object types within the group of subjects who shared a strong sense of organizational belongingness, and this finding corresponded to the results of the self-reported psychological ownership data.

These ERP findings have important implications. Our results provide neural evidence for the proposition that psychological ownership reflects both an affective and cognitive state (Shu and Peck, 2011). First, since P300 dissociated high from low self-relevance, as an index of the allocation of information-processing resources (Knyazev, 2013), psychologically owned stimuli presumably have preferential access to attention compared with other stimuli because of their greater psychological salience. As reported by Su et al. (2010) and Tacikowski and Nowicka (2010), the P300 component may index the evaluation of salient and motivationally relevant information. It has also been suggested that endowment effect over time can be partially explained by differential attentional focus and information seeking that differs based on one’s perspective (Ashby et al., 2012). Therefore, our findings indicate that as soon as an object is considered as “mine,” regardless of the state of its legal ownership, an individual’s cognitive relationship with the object alters. That is, the cognitive system prioritizes the representation of psychologically owned items based on the salient categorization and the relative personal meaning. Second, going beyond purely cognitive interpretations, P300 also seems to vary with the emotional value of the stimulus. According to Johnston et al. (1986), P300 was generated in response to stimuli with a personal emotional value. Tacikowski and Nowicka (2010) also revealed significantly enhanced P300 for emotionally charged representations as compared to neutral ones, suggesting that the ERP waveform is influenced by the emotional content of the experimental material. These two interpretations – seeing the P300 amplitude as an index of both attention and emotional arousal – are not mutually exclusive but could complement one another. As psychologically owned items often share meaningful associations with the self, these items could be treated as highly emotional. Thus, increased allocation of attentional resources and stronger emotional response to psychologically owned items may jointly contribute to a larger P300 amplitude (Johnson et al., 2002; Chiao et al., 2010). Such significantly enhanced P300 can also potentially explain other effects of psychological ownership, such as the tendency to assess our own objects as more attractive, more valuable and higher mnemonic advantage, for both self-relevant and ingroup-relevant cues (De Bortoli Vizioli et al., 2020). When psychologically owned object is presented, an enlarged deployment of cognitive resources is increased to achieve a better coupling between attention, memory, and decision-making in response (Sui and Humphreys, 2015).

From a theoretical perspective, the present study is the first to explore how organizational belongingness may impact perceived psychological ownership of organization-linked objects and the underlying neural dynamics. While most existing literature emphasizes the uniqueness of psychological ownership and its consequences on individual attitudes and behaviors (Karahanna et al., 2015), the role of motivational forces that serve to set individuals on their paths to psychological ownership is relatively neglected. We provide experimental evidence that the sense of organization belongingness and its association with home serve as a motive driving the emergence of psychological ownership over in-organization objects. Furthermore, although some neuroscientific research has considered the neural bases of psychological ownership (e.g., Turk et al., 2011; Kim and Johnson, 2012, 2014; Lockwood et al., 2018), the type of psychological ownership in these studies was transient and experimentally manipulated by associative-learning tasks. This study, however, provides much stronger evidence of the association between psychological ownership and the neural activities related to the cognition and emotion that lead to the emergence of psychological ownership. We demonstrate that psychological ownership is associated with increased attentional processing and a stronger emotional response in a more ecological context, as reflected by the P300 component. While the measurement of the psychological ownership construct has focused mostly on self-reported feelings and attitudes, there may exist the common method bias for linking psychological ownership with the measurement of its determinants and its consequences. By exploring the electrophysiological indicators as a tool for the measurement of psychological ownership, such common method bias can be addressed.

In sum, our findings demonstrate that psychological ownership can be elicited by marking the generic object with the name of the individual’s own organization. We also provide experimental evidence that the sense of organization belongingness plays a key role in the emergence of psychological ownership over in-organization objects. Moreover, the results show that psychological ownership, which has been portrayed as both an affective and cognitive state, can be indexed by the P300 component.

## Data Availability Statement

The raw data supporting the conclusions of this article will be made available by the authors, without undue reservation.

## Ethics Statement

The studies involving human participants were reviewed and approved by the Ethics Committee of Nankai University. The patients/participants provided their written informed consent to participate in this study.

## Author Contributions

WW, JP, DL, and JL conceived and designed the experiments. WW, JL, and XN performed the experiments. WW and GW analyzed the data. WW, JP, DL, GW, JL, and XN contributed to the writing of the manuscript. All authors contributed to the article and approved the submitted version.

## Funding

This work was supported by the National Social Science Foundation of China (grant numbers 20AZD044 and 18BDJ084), the National Natural Science Fund of China (grant number 71673152), Major Research Project of Humanities and Social Sciences of Shandong University (No. 21RWZD15), and Taishan Scholar Program of Shandong Province (No. tsqn201909013).

## Conflict of Interest

The authors declare that the research was conducted in the absence of any commercial or financial relationships that could be construed as a potential conflict of interest.

## Publisher’s Note

All claims expressed in this article are solely those of the authors and do not necessarily represent those of their affiliated organizations, or those of the publisher, the editors and the reviewers. Any product that may be evaluated in this article, or claim that may be made by its manufacturer, is not guaranteed or endorsed by the publisher.

## Footnotes

^1^Psychological ownership can also exist as a group-level phenomenon, which is depicted as “the collectively held sense that this target of ownership (or a piece of that target) is collectively ‘ours’” (Pierce and Jussila, 2011, p. 812).

^2^The ERP provides a non-invasive electrophysiological measure of real-time brain response, comprising a series of negative and positive voltage deflections which reflect different stages of cognition and vary based on extent of information processing (Macar and Vidal, 2004).

^3^Our results were robust when we included these 12 subjects either in HBG or LBG.

^4^According to the effect size of interaction effect in the first study, we used the G * Power 3.1 software, set Power to 95%, the alpha level of 0.05, and calculate the sample size in each group to 12.
